# Cryptosporidiosis in broiler chickens in Zhejiang Province, China: molecular characterization of oocysts detected in fecal samples

**DOI:** 10.1051/parasite/2014035

**Published:** 2014-07-31

**Authors:** Lengmei Wang, Xue Xue, Jianqiu Li, Qianjin Zhou, Yingchao Yu, Aifang Du

**Affiliations:** 1 Institute of Preventive Veterinary Medicine, College of Animal Sciences, Zhejiang University Hangzhou, Zhejiang 310058 PR China; 2 Hangzhou College of Commerce, Zhejiang Gongshang University Hangzhou, Zhejiang 310012 PR China; 3 Faculty of Life Science and Technology, Ningbo University Ningbo, Zhejiang 315211 PR China

**Keywords:** *Cryptosporidium*, Infection rate, China, Chicken, Epidemiology

## Abstract

*Cryptosporidium* is one of the most important parasites in poultry, and this pathogen can infect more than 30 avian species. The present study investigated the infection rate of *Cryptosporidium* among broiler chicken flocks. A total of 385 fecal samples from broiler chickens in 7 regions of Zhejiang Province collected from November 2010 to January 2012 were examined by microscopy. Thirty-eight (10%) samples were positive for *Cryptosporidium* infection, and 3 genotypes (*Cryptosporidium baileyi*, *Cryptosporidium meleagridis*, and avian genotype II) were identified by PCR and sequencing. A phylogenetic tree of the isolates was analyzed. These results suggest that cryptosporidiosis is widespread in poultry in Zhejiang Province, and is a potential threat to public health as well as the economy. This is the first report about the infection rate and molecular characterization of *Cryptosporidium* in broiler chickens in Zhejiang.

## Abstract (fr)

*Cryptosporidium* est l’un des parasites les plus importants de la volaille, et cet agent pathogène peut infecter plus de 30 espèces aviaires. La présente étude a examiné le taux d’infection de *Cryptosporidium* chez les poulets de chair. Un total de 385 échantillons fécaux de poulets de chair dans 7 régions de la province du Zhejiang, recueillis de novembre 2010 à janvier 2012, ont été examinés par microscopie. Trente-huit (10 %) étaient positifs pour l’infection à *Cryptosporidium*, et 3 génotypes (*Cryptosporidium baileyi*, *Cryptosporidium meleagridis*, génotype aviaire II) ont été identifiés par PCR et séquençage. Un arbre phylogénétique des isolats a été analysé. Ces résultats suggèrent que la cryptosporidiose est répandue chez les volailles dans la province du Zhejiang, et est une menace potentielle pour la santé publique ainsi que l’économie. Ceci est le premier rapport sur le taux d’infection et la caractérisation moléculaire de *Cryptosporidium* dans les poulets de chair dans le Zhejiang.

## Introduction

Cryptosporidiosis is caused by *Cryptosporidium*, which has a broad range of hosts and worldwide distribution. The *Cryptosporidium* species can infect human beings as well as other animals; it is mainly located in the epithelial cells of the gastrointestinal tract, and is likely in the respiratory tract [7], particularly in the case of infection by *C. meleagridis*, a bird species. The main clinical manifestation is persistent diarrhea, and respiratory disturbances can occasionally be observed [5, 12].

*Cryptosporidium* is one of the most common and important parasites in poultry, and this pathogen can infect more than 30 avian species. Traditionally, three different species of *Cryptosporidium* (*C. baileyi*, *C. meleagridis*, and *C. galli*) were considered to be the major species in birds [4, 6, 17, 20, 23, 30]. Recently, more than 11 novel genotypes have been described which are genetically different from the above three species by means of molecular analysis, including avian genotypes (I–V), goose genotypes (I–IV), the Eurasian woodcock genotype and the black duck genotype [1, 9, 14–17, 20, 31]. *C. meleagridis*, *C. parvum*, *and C. hominis* carried by poultry may infect human beings through the oocysts in contaminated water or vegetables eaten uncooked [1, 13, 20, 31, 32]. Considering that the possibility of exposure to *Cryptosporidium* of human beings still exists, the prevention of Cryptosporidiosis is important for public health [10].

Up to now, there is little information regarding the distribution of *Cryptosporidium* spp. in Zhejiang Province, and also limited data about its molecular characterization in poultry, particularly in chickens. The aim of the present study was to estimate the infection rate of *Cryptosporidium* in poultry in Zhejiang Province, and to determine the genotypes in the area.

## Materials and methods

### Sample collection

Three hundred and eighty-five fecal samples were randomly collected from broiler chickens that were around 90 days old in seven regions (Hangzhou, Huzhou, Jiaxing, Jinhua, Ningbo, Quzhou, and Shaoxing) in Zhejiang Province during the period from November 2010 to January 2012. Chickens were reared in steel cages; each contained 5 ~ 7 birds whose feces were collected on the tray under the cages gathered as a mixture. Therefore, the feces in one cage were considered as one sample, collected by the use of disposable plastic gloves, marked with the region name, serial number, and collection date. The sampling process was conducted to collect the fresh droppings to the best of our ability. The weight of each sample was approximately equal to about 50 g. Samples were kept in ice boxes until they were transported to the laboratory, then stored in the refrigerator at 4 °C and processed as soon as possible.

### Microscopy detection

Samples were handled by Sheather’s sugar flotation method as previously described by Huber et al. [8], and *Cryptosporidium* oocysts were examined by optical microscopy observation under 400× magnification based on the shape of oocysts and the shape index measured. Subsequently, the positive samples containing oocysts were stored in 2.5% potassium dichromate and kept at 4 °C until DNA extraction.

### DNA extraction

Oocysts in positive samples were purified by discontinuous sucrose density gradient centrifugation [3]. For genomic DNA extraction, 100 μL of suspension liquid containing oocysts was frozen-thawed for 5 min in liquid nitrogen and then kept in a 65 °C water bath kettle for 5 min. The process was repeated three times, then the treated samples were centrifuged at 12 000 × g for 5 min. The genomic DNA was extracted using a Genomic DNA Extraction Kit (TaKaRa Biotechnology (Dalian) Co. Ltd., Dalian, China) in accordance with the manufacturer’s instructions, and kept at −20 °C until detected by the PCR method.

### Nested-PCR amplification and sequencing

A nested PCR was done in order to amplify a fragment of approximately 830 bp [30]. Then, the secondary purified PCR product was sequenced (BGI sequencing) to confirm the species/genotype identification.

### *Cryptosporidium* genotyping and phylogenetic analysis

The acquired sequences were submitted to a BLAST search (http://blast.ncbi.nlm.nih.gov/Blast.cgi) to initially define the species/genotypes and to confirm the high similarity and homology with other known sequences of *Cryptosporidium* spp. in GenBank. All sequences were multiple-aligned and analyzed by Bioedit and MEGA 4.0 software (http://www.mbio.ncsu.edu/BioEdit/bioedit.html and http://www.megasoftware.net/). A neighbor-joining cladogram was built using MEGA 4.0. To assess the reliability of this tree, bootstrap analysis was done with 1000 replicates using the Kimura 2-parameter logarithm.

The partial 18S rRNA nucleotide sequences obtained in this study have been deposited in the GenBank database under accession numbers JX548291–JX548300.

### Statistical analysis

The infection rate of *Cryptosporidium* was analyzed using the chi-square test. Differences were considered significant when *P* < 0.05.

## Results

### Infection rate of *Cryptosporidium* in Zhejiang Province

Of the 385 fecal samples from broiler chickens examined in this study, 38 samples were positive, and the overall infection rate of *Cryptosporidium* was 9.9%. The infection rate of *Cryptosporidium* varied among the seven regions. The highest infection rate was 16.9% in Huzhou, followed by 16.7% in Hangzhou, 12.1% in Ningbo, 7.7% in Shaoxing, 5.3% in Jiaxing, 4.0% in Quzhou, and 2.3% in Jinhua (Table 1). Significant statistical differences were not found in relation to the geographical provenance of chickens and infection rates in each region.

**Table 1.** Infection rate of *Cryptosporidium* in different source supplied for the native chicken.

Region	No. of cages examined	No. of cages *Cryptosporidium* positive	Infection rate (%)	*Cryptosporidium* species/genotypes (No. of isolates)
Hangzhou	66	11	16.7	*C. baileyi* (10), *C. meleagridis* (1)
Huzhou	59	10	16.9	*C. baileyi* (7), *C. meleagridis* (1), Avian genotype II (2)
Shaoxing	52	4	7.70	*C. baileyi* (4)
Ningbo	58	7	12.1	*C. baileyi* (6), Avian genotype II (1)
Jiaxing	57	3	5.26	*C. baileyi* (3)
Jinhua	43	1	2.33	*C. baileyi* (1)
Quzhou	50	2	4.00	*C. baileyi* (2)
Total	385	38	9.87	*C. baileyi* (33), *C. meleagridis* (2), Avian genotype II (3)

### Distribution of *Cryptosporidium* species

**Figure 1.**
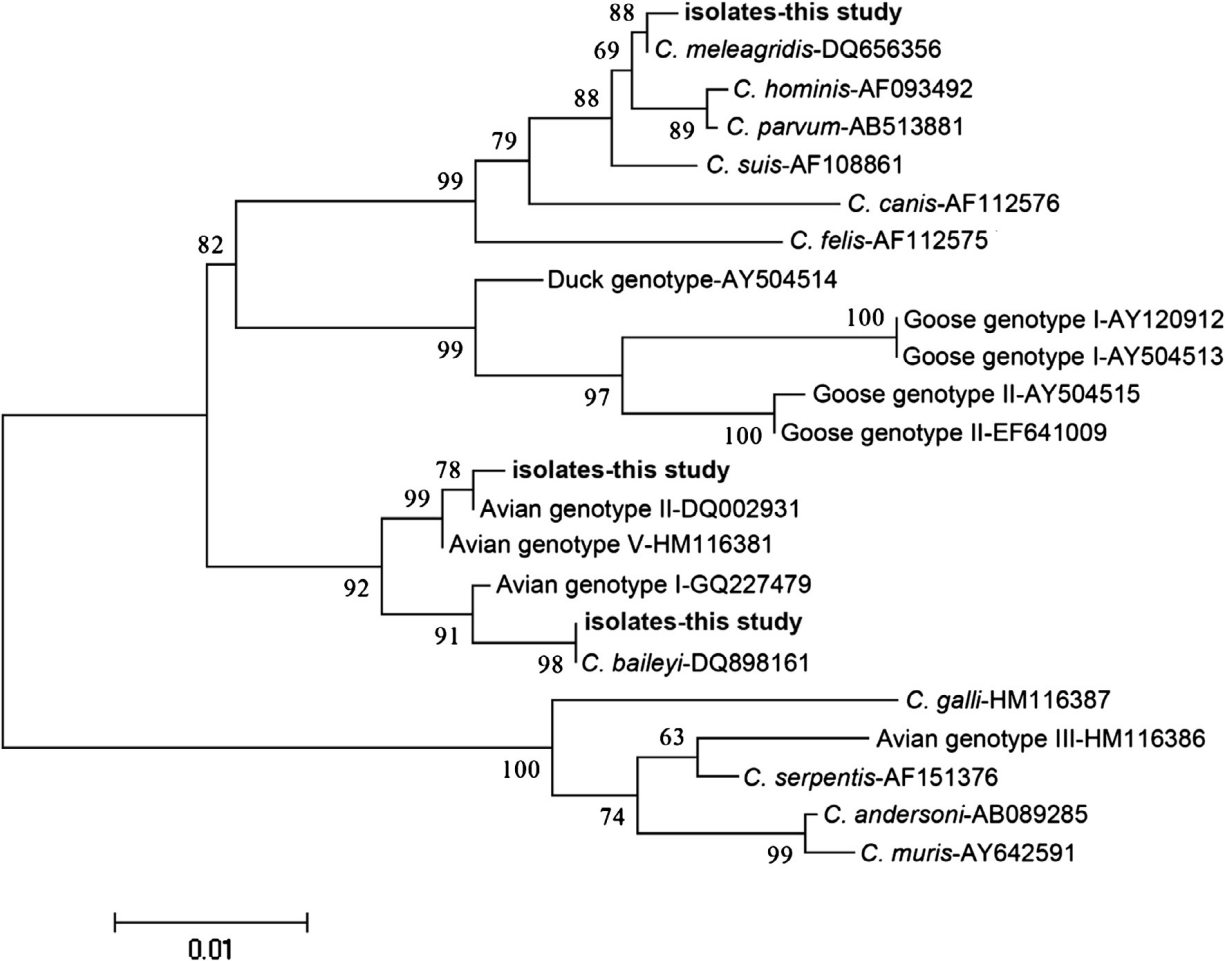
Phylogenetic tree of *Cryptosporidium* spp. built by the neighbor-joining method using Mega 4.0, based on 18S rRNA. To assess the reliability of this tree, bootstrap analysis was done with 1000 replicates.

Two species and one genotype, including *C. baileyi*, *C. meleagridis*, and the avian genotype II, were identified through morphological observation and sequencing. *C. baileyi* was widely distributed in the seven areas. 33 of 38 positive samples were for *C. baileyi* infection (86.8%), only two samples for *C. meleagridis* infection (5.3%), and three samples for avian genotype II infection (7.9%). Moreover, the average size of oocysts for *C. baileyi* was 5.96 μm × 4.73 μm by measuring 35 oocysts, for *C. meleagridis* it was 4.96 μm × 4.15 μm, and for avian genotype II it was 6.02 μm × 5.47 μm. The neighbor-joining among these species/genotypes and other previously described species and genotypes of *Cryptosporidium* is shown in Figure 1.

### Sequence and phylogenetic analysis

The 18S rRNA gene of *C. baileyi* isolates showed high sequence homology with each other by direct sequence alignment, and exhibited 99% sequence similarity with a GenBank sequence of quail origin (DQ898161). Two *C. meleagridis* isolates showed 99.5% sequence similarity with a GenBank sequence from Iran (DQ656356). Three avian genotype II isolates showed 99.2% sequence similarity with a GenBank sequence (DQ002931).

## Discussion

*Cryptosporidium* is widespread around the world except Antarctica, and is most common in developing countries and less developed countries [22]. Thus far, there are few data available about the infection rate of *Cryptosporidium* in poultry, especially in chickens in China. It was reported that the infection rate of *Cryptosporidium* was 3.4% in Henan and 23.8% in Shandong, China [26, 27]. Moreover, the rate was 3.75% in the Garmsar region, Iran, and 4.5% in Tunisia [21, 24]. It was clear that the overall rate in this study was much higher than in other countries. We speculate that this difference may be attributed to the different approaches in the sample collection. In our study, the fecal sample was collected from a pool of animals; thus, it is possible to find a higher rate of infection when compared with individual samples. Moreover, the various environmental factors and host species may also account for the difference [2]. The differences between different studies may be due to various factors such as seasonality, the age of chickens, the breeding environment, etc. Given the similar climate of the regions in Zhejiang Province, the interregional variation in infection rates observed could be due to the nutrient levels, breeding conditions and the density of animals [11–25].

In China, the infection rate of *Cryptosporidium* derived from poultry has been reported so far in almost 10 provinces. However, most researchers were focused on the prevalence in the region through traditional morphological observation, rather than identifying the species or genotypes of the *Cryptosporidium* by modern molecular biology approaches. One exception is Henan Province. In more recent studies, the prevalence of *Cryptosporidium* spp. has been reported in detail in quails, chickens, Pekin ducks, pet birds, and ostriches in Henan, and most species have been identified as *C. baileyi* and *C. meleagridis* [18, 27–29]. The epidemiological investigation of *Cryptosporidium* in poultry in Zhejiang Province was carried out in our research.

Among the three *Cryptosporidium* species/genotypes identified in this study, *C. baileyi* was the primary species of the broiler chickens, for the reason that it was not only widely distributed in the seven areas, but also had the highest percentage of positive samples (33/38). Thus, the phenomenon of *C. baileyi* infection in broiler chickens in this study confirmed the following views. *C. baileyi*, which is the most common species in poultry, has more than 20 kinds of hosts including chickens [19]. In addition, studies in China showed that most cases in chickens were diagnosed as *C. baileyi* infections, which can slow down the growth rate in broilers and decrease egg production of layer chickens. Because the poultry industry plays a critical role in the agricultural economy in China, it is essential to pay sufficient attention to this pathogen. Only two isolates were identified as *C. meleagridis* in our investigation. Meanwhile, *C. meleagridis* had extensive hosts too, and it could cause deadly diseases in immunocompromised persons [19]. People could be infected with *C. meleagridis* by potential zoonotic transmission when the chickens carry the pathogen. Therefore, it has a great influence on public health. Three isolates were identified as avian genotype II in the present study. Avian genotype II was first described in turkey and was reported in parrot in the same year [14, 16]. Afterward, avian genotype II was found in ostriches [17]. Recently, chickens have also appeared to be a new host for avian genotype II. Perhaps we could make a bold conjecture that avian genotype II also has a range of hosts like *C. baileyi* and *C. meleagridis*. In addition, according to the phylogenetic analyses of 18S rRNA, together with the analysis based on actin and HSP70 in a previous study by Abe and Makino [1], it is further assumed that avian genotype II has a close relationship with avian genotype V at a genetic level. The cladogram in the present study proved the above points, which demonstrated the reliability of the tree.

In conclusion, this is the first report about the infection rate and molecular characterization of *Cryptosporidium* in broiler chickens in Zhejiang. The results suggest that *Cryptosporidium* is widespread in poultry in Zhejiang Province, and may pose a potential threat to public health as well as the economy.

## References

[1] Abe N, Makino I. 2010 Multilocus genotypic analysis of *Cryptosporidium* isolates from cockatiels, Japan. Parasitology Research, 106, 1491–1497 20339870 10.1007/s00436-010-1810-5

[2] Amer S, Zidan S, Adamu H, Ye J, Roellig D, Xiao L, Feng Y. 2013 Prevalence and characterization of *Cryptosporidium* spp. in dairy cattle in Nile River delta provinces, Egypt. Experimental Parasitology, 135, 518–523 24036320 10.1016/j.exppara.2013.09.002

[3] Arrowood MJ, Donaldson K. 1996 Improved purification methods for calf-derived *Cryptosporidium parvum* oocysts using discontinuous sucrose and cesium chloride gradients. Journal of Eukaryotic Microbiology, 43, S89–S89 10.1111/j.1550-7408.1996.tb05015.x 8822880

[4] Current WL, Upton SJ, Haynes TB. 1986 The life cycle of *Cryptosporidium baileyi* n. sp. (Apicomplexa, Cryptosporidiidae) infecting chickens. Journal of Protozoology, 33, 289–296 3735157 10.1111/j.1550-7408.1986.tb05608.x

[5] Das G, Changkija B, Sarkar S, Das P. 2011 Genotyping of *Cryptosporidium parvum* isolates in bovine population in Kolkata and characterization of new bovine genotypes. Research in Veterinary Science, 91, 246–250 21316723 10.1016/j.rvsc.2011.01.003

[6] Fayer R. 2010 Taxonomy and species delimitation in *Cryptosporidium*. Experimental Parasitology, 124, 90–97 19303009 10.1016/j.exppara.2009.03.005

[7] Gomes RS, Huber F, da Silva S, do Bomfim TC. 2012 *Cryptosporidium* spp. parasitize exotic birds that are commercialized in markets, commercial aviaries, and pet shops. Parasitology Research, 110, 1363–1370 21922240 10.1007/s00436-011-2636-5

[8] Huber F, Bomfim TC, Gomes RS. 2005 Comparison between natural infection by *Cryptosporidium* sp., *Giardia* sp. in dogs in two living situations in the West Zone of the municipality of Rio de Janeiro. Veterinary Parasitology, 130, 69–72 15893071 10.1016/j.vetpar.2005.03.012

[9] Jellison KL, Distel DL, Hemond HF, Schauer DB. 2004 Phylogenetic analysis of the hypervariable region of the 18S rRNA gene of *Cryptosporidium* oocysts in feces of Canada geese (*Branta canadensis*): evidence for five novel genotypes. Applied and Environmental Microbiology, 70, 452–458 14711674 10.1128/AEM.70.1.452-458.2004 PMC321269

[10] Karanis P, Kourenti C, Smith H. 2007 Waterborne transmission of protozoan parasites: a worldwide review of outbreaks and lessons learnt. Journal of Water and Health, 5, 1–38 17402277 10.2166/wh.2006.002

[11] Lai M, Zhou RQ, Huang HC, Hu SJ. 2011 Prevalence and risk factors associated with intestinal parasites in pigs in Chongqing, China. Research in Veterinary Science, 91, e121–e124 21349561 10.1016/j.rvsc.2011.01.025

[12] Langsley G, van Noort V, Carret C, Meissner M, de Villiers EP, Bishop R, Pain A. 2008 Comparative genomics of the Rab protein family in Apicomplexan parasites. Microbes and Infection, 10, 462–470 18468471 10.1016/j.micinf.2008.01.017 PMC3317772

[13] Majewska AC, Graczyk TK, Slodkowicz-Kowalska A, Tamang L, Jedrzejewski S, Zduniak P, Solarczyk P, Nowosad A, Nowosad P. 2009 The role of free-ranging, captive, and domestic birds of Western Poland in environmental contamination with *Cryptosporidium parvum* oocysts and *Giardia lamblia* cysts. Parasitology Research, 104, 1093–1099 19050920 10.1007/s00436-008-1293-9

[14] Meireles MV, Soares RM, dos Santos MM, Gennari SM. 2006 Biological studies and molecular characterization of a *Cryptosporidium isolate* from ostriches (*Struthio camelus*). Journal of Parasitology, 92, 623–626 16884009 10.1645/0022-3395(2006)92[623:BSAMCO]2.0.CO;2

[15] Morgan UM, Monis PT, Xiao L, Limor J, Sulaiman I, Raidal S, O’Donoghue P, Gasser R, Murray A, Fayer R, Blagburn BL, Lal AA, Thompson RC. 2001 Molecular and phylogenetic characterisation of *Cryptosporidium* from birds. International Journal for Parasitology, 31, 289–296 11226456 10.1016/s0020-7519(00)00164-8

[16] Ng J, Pavlasek I, Ryan U. 2006 Identification of novel *Cryptosporidium* genotypes from avian hosts. Applied and Environmental Microbiology, 72, 7548–7553 17028234 10.1128/AEM.01352-06 PMC1694252

[17] Nguyen ST, Fukuda Y, Tada C, Huynh VV, Nguyen DT, Nakai Y. 2013 Prevalence and molecular characterization of *Cryptosporidium* in ostriches (*Struthio camelus*) on a farm in central Vietnam. Experimental Parasitology, 133, 8–11 23142549 10.1016/j.exppara.2012.10.010

[18] Qi M, Wang R, Ning C, Li X, Zhang L, Jian F, Sun Y, Xiao L. 2011 *Cryptosporidium* spp. in pet birds: genetic diversity and potential public health significance. Experimental Parasitology, 128, 336–340 21557938 10.1016/j.exppara.2011.04.003

[19] Ryan U. 2010 *Cryptosporidium* in birds, fish and amphibians. Experimental Parasitology, 124, 113–120 19545515 10.1016/j.exppara.2009.02.002

[20] Ryan UM, Xiao L, Read C, Sulaiman IM, Monis P, Lal AA, Fayer R, Pavlasek I. 2003 A redescription of *Cryptosporidium galli* Pavlasek, 1999 (Apicomplexa: Cryptosporidiidae) from birds. Journal of Parasitology, 89, 809–813 14533694 10.1645/GE-74RI

[21] Shemshadi B, Rangbar Bahadori S, Mozafari A. 2010 Study on cryptosporidiosis incidence in broilers in Garmsar region, Iran. Comparative Clinical Pathology, 20, 143–149

[22] Shirley D, Moonah S, Kotloff K. 2012 Burden of disease from cryptosporidiosis. Current Opinion in Infectious Diseases, 25, 555–563 22907279 10.1097/QCO.0b013e328357e569 PMC4465599

[23] Slavin D. 1955 *Cryptosporidium meleagridis* (sp. nov.). Journal of Comparative Pathology, 65, 262–266 13242675 10.1016/s0368-1742(55)80025-2

[24] Soltane R, Guyot K, Dei-Cas E, Ayadi A. 2007 Prevalence of *Cryptosporidium* spp. (Eucoccidiorida: Cryptosporiidae) in seven species of farm animals in Tunisia. Parasite, 14, 335–338 18225423 10.1051/parasite/2007144335

[25] Taylor M, Webster K. 1998 Recent advances in the diagnosis in livestock of *Cryptosporidium*, *Toxoplasma*, *Giardia* and other protozoa of veterinary importance. Research in Veterinary Science, 65, 183–193 9915141 10.1016/S0034-5288(98)90141-2 PMC7131700

[26] Ti-sen X. 2009 Epidemiological investigation of *Cryptosporidium* from poultry in Shandong province. Chinese Journal of Veterinary Medicine, 4, 3–5

[27] Wang R, Jian F, Sun Y, Hu Q, Zhu J, Wang F, Ning C, Zhang L, Xiao L. 2010 Large-scale survey of *Cryptosporidium* spp. in chickens and Pekin ducks (*Anas platyrhynchos*) in Henan, China: prevalence and molecular characterization. Avian Pathology, 39, 447–451 21154053 10.1080/03079457.2010.518314

[28] Wang R, Qi M, Zhu J, Sun D, Ning C, Zhao J, Zhang L, Xiao L. 2011 Prevalence of *Cryptosporidium baileyi* in ostriches (*Struthio camelus*) in Zhengzhou, China. Veterinary Parasitology, 175, 151–154 21030152 10.1016/j.vetpar.2010.10.005

[29] Wang R, Wang F, Zhao J, Qi M, Ning C, Zhang L, Xiao L. 2012 *Cryptosporidium* spp. in quails (*Coturnix coturnix japonica*) in Henan, China: Molecular characterization and public health significance. Veterinary Parasitology, 187, 534–537 22377447 10.1016/j.vetpar.2012.02.002

[30] Xiao L, Fayer R, Ryan U, Upton SJ. 2004 *Cryptosporidium* taxonomy: recent advances and implications for public health. Clinical Microbiology Reviews, 17, 72–97 14726456 10.1128/CMR.17.1.72-97.2004 PMC321466

[31] Zhou L, Kassa H, Tischler ML, Xiao L. 2004 Host-adapted *Cryptosporidium* spp. in Canada geese (*Branta canadensis*). Applied and Environmental Microbiology, 70, 4211–4215 15240303 10.1128/AEM.70.7.4211-4215.2004 PMC444829

[32] Zhou P, Chen N, Zhang R-L, Lin R-Q, Zhu X-Q. 2008 Food-borne parasitic zoonoses in China: perspective for control. Trends in Parasitology, 24, 190–196 18314393 10.1016/j.pt.2008.01.001

